# VSD repair in patients after previous failed pulmonary artery banding

**DOI:** 10.1186/1749-8090-8-S1-P94

**Published:** 2013-09-11

**Authors:** OS Golovenko, WM Novick, SO Siromakha, VV Lazoryshynets

**Affiliations:** 1 Department of Congenital Heart Defects Surgery, Amosov National Institute of Cardiovascular Surgery, Kyiv, Ukraine; 2 Departments of Surgery and Pediatrics, University of Tennessee Health Sciences Center, Memphis, TN, USA

## Background

In the current surgical era PA banding is not indicated as palliation or stage procedure for patients with large VSD and severe PAH. We have reviewed our institutions experience in patients undergoing VSD repair with previously failed PA banding. Failure of PA banding was defined as pulmonary to aortic systolic pressure ratio (PASPR) > 0.8 in period of at least 6 months after palliation.

## Methods

8 patients (median age 5,6±4,5 years) with a large VSD and mean PASPR 0.89±0.7 had VSD repair done (2 pts using double patch flap valve technique) between 1998-2012. The period from previous palliation ranged from 9 months to 13 years. All patients underwent pre-operative catheterization using oxygen provocation. Mean PVR was 10.2±6.1 Wood units (WU) and 6 of 8 pts were responsible to vasoreactivity test. Follow-up was conducted on all surviving patients.

## Results

The early mortality rate was 12.5% (1/8). It was 14 years old ccTGA patient with preop PVR 14.6 WU and negative response to oxygen provocation. There were no late deaths at last follow-up (from 1 to 12 years). One patient with preop PVR 9.0 WU and negative oxygen test developed near to systemic pulmonary artery pressure one year after operation. 6 (75%) patients showed significant drop of PASPR < 0.5 (0.41±0.09).

## Conclusion

Successful VSD repair can be performed safely with an acceptable mortality results even if previous PA banding was failed. The most important criteria of operability can be defined using vasodilators provocation test during catheterization. Patients with negative results could be considered as inoperable.

